# Heterogeneity of Group 2 Innate Lymphoid Cells Defines Their Pleiotropic Roles in Cancer, Obesity, and Cardiovascular Diseases

**DOI:** 10.3389/fimmu.2022.939378

**Published:** 2022-06-29

**Authors:** Masashi Ikutani, Susumu Nakae

**Affiliations:** ^1^Laboratory of Immunology, Program of Food and AgriLife Science, Graduate School of Integrated Sciences for Life, Hiroshima University, Higashi-Hiroshima, Japan; ^2^Precursory Research for Embryonic Science and Technology, Japan Science and Technology Agency, Saitama, Japan

**Keywords:** group 2 innate lymphoid cell, interleukin-5, interleukin-33, eosinophil, anti-tumor immunity, obesity, cardiovascular disease

## Abstract

Group 2 innate lymphoid cells (ILC2s) are typically known for their ability to respond rapidly to parasitic infections and play a pivotal role in the development of certain allergic disorders. ILC2s produce cytokines such as Interleukin (IL)-5 and IL-13 similar to the type 2 T helper (Th2) cells. Recent findings have highlighted that ILC2s, together with IL-33 and eosinophils, participate in a considerably broad range of physiological roles such as anti-tumor immunity, metabolic regulation, and vascular disorders. Therefore, the focus of the ILC2 study has been extended from conventional Th2 responses to these unexplored areas of research. However, disease outcomes accompanied by ILC2 activities are paradoxical mostly in tumor immunity requiring further investigations. Although various environmental factors that direct the development, activation, and localization of ILC2s have been studied, IL-33/ILC2/eosinophil axis is presumably central in a multitude of inflammatory conditions and has guided the research in ILC2 biology. With a particular focus on this axis, we discuss ILC2s across different diseases.

## Introduction

Recent expansion in our understanding of innate lymphoid cells (ILCs) began with several epoch‐making reports in 2010 (1–4). The ILCs were originally indicated as interleukin (IL)-25 responsive non-B/non-T lymphocytes (5). ILCs are classified into five distinct cell populations based on their characteristics, including the profile of cytokines produced and the key transcription factors involved in their major immunological functions. These are the natural killer (NK) cells, group 1 ILC (ILC1), ILC2, ILC3, and lymphoid tissue inducer (LTi) cells (6). This classification should be observed with caution because ILCs possess a unique plastic ability to adapt to the surrounding milieu and can undergo transdifferentiation into another group of ILCs (7–9). ILC2s are tissue-resident cells (10), preferentially inhabiting the mucosal organs such as lung and intestine, and display tissue-specific transcriptional features influenced by the surrounding environment (11). The mucosal surface is the first line of defense against infectious pathogens; hence, ILC2s inherently display a prompt response through the secretion of IL-5 and IL-13. Unlike T cells, ILC2s lack antigen-specific receptors and instead express receptors for epithelial-derived cytokines such as thymic stromal lymphopoietin (TSLP), IL-25, and IL-33, thereby ensuring signal recognition from exogenous agents. ILC2s not only participate in acute responses but also in the subsequent antigen-specific adaptive immune responses in cooperation with type 2 T helper (Th2) cells (12, 13). In addition to Th2 cytokines, ILC2s in the lung were found to produce IL-10 (14–16) and IL-10-producing ILCs, so-called ILCreg, have been reported in the intestine (17). Interestingly, like other antigen-presenting cells, ILC2s communicate through major histocompatibility complex class II molecules to activate acquired immune response (18). Thus, ILC2s provide a link between innate and acquired immunity (19).

ILC2s are involved in various immunological disorders and host defense (20). Asthma is a chronic airway inflammatory disease and one of the best-characterized allergic disorders associated with ILC2s (21, 22). ILC2s serve to establish predominant Th2 inflammation synergistically and/or competitively by interacting with other ILC subsets and immune cells (23). ILC2s in respiratory diseases are also evident in humans (24). To eliminate invading parasites, ILC2s mediate Th2 immune response in collaboration with adaptive Th2 cells (25, 26). In anti-viral immunity, although ILC2s exacerbate airway hyperreactivity through IL-13 production (27), they contribute to tissue repair by producing a wound-healing protein, amphiregulin (28). In most cases, IL-33 is considered a central cytokine for such ILC2-mediated immune responses.

Although the functions of IL-33 in allergies are well known (29, 30), the focus has currently shifted to its role in cancer (31–33) and cardiovascular diseases (34–36). IL-33 is one of the most effective cytokines for regulating ILC2s. In a steady state, IL-33 resides in the nucleus and is released by necrotic cells within damaged tissue (29, 37). When a tissue is injured/infected by pathogens, IL-33 acts by alarming the immune cells in the vicinity to mediate immune responses, and is thus called an “alarmin” or damage-associated molecular pattern. The IL-33 receptor comprises ST2 (IL-1 receptor-like 1) and IL-1 receptor accessory protein (38–40) which is expressed on various immune cells (41, 42). The binding of IL-33 to ST2 on the cell surface ensures Th2 responses, whereas soluble ST2 (sST2) in circulation inhibits excess IL-33-mediated responses and protects against disease development (29). In an allergic inflammation, platelets act as reservoirs and suppliers of IL-33 (43) and are capable of boosting ILC2 activities through direct interaction (44). In the lung tissue, platelets are supplied by the resident megakaryocytes (45) and may participate in regulation of ILC2 function.

IL-5 is a homodimeric cytokine and its engagement with its receptor, comprising an IL-5Rα and a common β-chain, plays critical roles in eosinophil biology starting from the early phase of its ontogeny in bone marrow (46, 47). Eosinophils store mediators such as major basic protein (MBP) in granules and are involved in both health and disease (48, 49). Genetic blockade of IL-5 signaling results in severe defects in eosinophil regulation (50, 51), and therefore treatments with anti-IL-5 or anti-IL-5Rα monoclonal antibodies (mAb) have been promising in patients with severe eosinophilic asthma (52–54).

In this review, we will discuss recent findings describing ILC2s in different types of disorders, such as cancer, obesity, and cardiovascular diseases. These findings suggest that roles of ILC2s are pleiotropic and diverse, largely depending on the surrounding environment. An ILC2-targeted therapeutic approach effective for one disease might be deleterious for another. This highlights the requirement for a detailed investigation and verification of the association and mechanisms of ILC2s.

### Contradictory Roles of ILC2s in Tumor Immunity

Recent findings have shed light on both anti- and pro-tumor activities of ILC2s (55–60). The anti-tumorigenic activity of ILC2s appears to be largely dependent on the requirement of eosinophils at the site of malignancy. Histological evidence for the involvement of eosinophils in human cancers exists (61–63), however, the findings are controversial (64, 65). The number of infiltrated eosinophils in colonic or colorectal carcinomas significantly correlates with improved prognosis (63, 66–70). Conversely, in cervical cancer (71), nasopharyngeal carcinoma (72), and lymph node metastasis or lymphatic invasion (73), eosinophils were associated with unfavorable prognoses. In addition to the direct cytotoxic activity of the granules containing MBP on tumor cells (74, 75), eosinophils in tumor microenvironment (TME), when activated by interferon (IFN)-γ and tumor necrosis factor (TNF)-α efficiently promote mobilization of CD8^+^ cytotoxic T cells from circulation (76). Eosinophils, however, display functional and phenotypical heterogeneity and their influence seems to rely on tumor types, TME, and cancer stages (64).

Involvement of IL-5-producing ILC2s in antitumorigenic activities was reported using an IL-5 reporter mouse (77), wherein lung ILC2s were required to retain sufficient number of eosinophils against tumor metastasis, and a blockade of IL-5 signaling resulted in an increased B16F10 metastasis. This is supported by the findings from a study that included three groups of mice deficient in C-C motif chemokine ligand 11 (CCL11), both CCL11 and IL-5, and eosinophils, respectively; all the three groups of mice exhibited increased tumor growth in chemically-induced fibrosarcoma (78). Antitumorigenic ILC2s are primed by their environment modulated by IL-33 (31–33). Mice inoculated with IL-33-expressing tumor cell lines, including EL4, CT26, and B16F10, resulted in a substantial expansion of intertumoral ILC2, which inhibited tumor growth and induced apoptosis of tumor cells through the production of CXC chemokine receptor 2 ligands (79). IL-33-expressing A9, a murine lung tumor cell line, was also reported to augment the antimetastatic functions of ILC2s (80). Although ILC2s were not investigated in mice administered with IL-33, tumor growth and metastasis were inhibited *via* eosinophil recruitment (81). Mice deficient in ILC2s failed to control the incidence of experimentally induced colorectal cancers, whereas ILC2 infiltration correlated with better overall survival of patients with colorectal cancers (82). TME induces the expression of programmed cell death-1 (PD-1) on CD8^+^ T cells as well as ILC2s, which results in the inhibition of cytokine production from these cells. Importantly, a blockade of PD-1 on the surface of ILC2s leads to revival of their antitumorigenic properties (83, 84). Interestingly, both serum IL-5 and IFN-γ levels are useful in predicting the efficacy of anti-PD1 mAb treatment in patients with non-small-cell lung and gastric cancer (85).

Accumulating evidence has also suggested pro-tumorigenic roles of ILC2s. In contrast to the previous study (77), IL-5 was reported to facilitate tumor metastasis (86). Additionally, IL-5 was suggested to enhance the migration of bladder cancer cells (87), and esophageal squamous cell carcinoma (88) in humans. Furthermore, IL-5 enhanced metastasis of breast cancer cells in obese mice (89). Consistent with these reports, ILC2s facilitated tumor metastasis in IL-33-treated animals by limiting cytotoxic activity of NK cells (90). Moreover, IL-13 derived from ILC2s promoted differentiation of myeloid-derived suppressor cells and were pro-tumorigenic in acute promyelocytic leukemia (91), bladder cancer recurrence (92), and metastasis of breast cancer (93).

Roles of ILC2s, eosinophils and IL-33 in tumor immunity show contrasting results, which poses a difficulty in understanding the distinct roles of these players in deciding the fate of tumor cells. However, the possibility of environmental cues as a key determinant for ILC2s to be antitumorigenic or pro-tumorigenic can be envisaged. For instance, lactic acid from tumor cells is pro-tumorigenic (94) whereas higher levels of IL-33 in TME are shown to induce antitumorigenic activities of ILC2s (81). This suggests that an assessment of the regulation of ILC2s by TME is essential for therapeutic intervention.

### Anti-Inflammatory and Thermogenic Roles of ILC2 in Obesity

Obesity is a highly prevalent condition worldwide in which excess fat accumulates in the body. It is often associated with type 2 diabetes, high blood pressure, hyperlipidemia, and cardiovascular diseases (95). Apart from the roles of ILC2s in typical Th2 immune responses, they also contribute to homeostatic and metabolic regulation in adipose tissues (96, 97). Adipose tissues are categorized into white, brown, and beige. In comparison to white, beige and brown adipose tissues display higher and the highest thermogenic activity, respectively, and are thus specialized in generating heat. Initially, eosinophils were demonstrated to be the major IL-4-producing cells in white adipose tissue involved in inducing anti-inflammatory M2 macrophages (98) which prevents weight gain. Furthermore, ILC2s in adipose tissues were the major sources of IL-5 and IL-13 and recruited eosinophils to produce an anti-obese environment (99). Conversely, ILC2s in the small intestine were reported to induce obesity through the production of IL-2 (100), indicating the importance of the interplay between distal organs.

ILC2s also directly regulate adipocytes and participate in thermogenesis (101, 102). Adipose ILC2s promote beiging, conversion from white to beige, through production of methionine-enkephalin peptide, which can directly affect the adipocytes and upregulate the expression of uncoupling protein 1 (101), which was brought about by IL-33 (103). In response to cold stimuli, ILC2s are responsible for proliferation of platelet-derived growth factor receptors (PDGFR)-α^+^ adipocyte progenitors and subsequent differentiation to beige adipocytes (102). IL-13 from ILC2s and/or IL-4 from eosinophils have been shown to stimulate PDGFRα^+^ progenitors through their surface IL-4R.

Cell-cell interaction is important for the activation of adipose ILC2s. Both glucocorticoid-induced TNF receptor (104) and death receptor 3 (105) belong to the TNFR superfamily and are expressed on adipose ILC2s. Post ligand binding, ILC2s accelerate the production of IL-5 and IL-13 and improve glucose tolerance and insulin sensitivity, demonstrating their potential to be used in type 2 diabetes therapy. In contrast, IL-33 in the presence of TNF-α in obese conditions upregulates PD-1 expression on adipose ILC2s and limits their production of IL-5 and IL-13 (106). Recently, regulation of ILC2s by sympathetic nerves *via* adipose mesenchymal stromal cells was observed (107). Elucidation of the precise regulatory mechanism and knowledge on the specific activators of adipose ILC2s will aid in therapy for obesity or type 2 diabetes.

### Reparative Roles of ILC2s in Cardiac Dysfunction

ILC2s are involved in healing cardiac tissue with cooperation from various types of immune cells to recover and regenerate cardiac tissue damage caused by myocardial infarction (MI) (108). ST2 is expressed on cardiomyocytes, and levels of sST2 in serum from animals and humans were elevated after MI (109). Therefore, IL-33 being the only known ligand of ST2 (38), its role in cardiovascular and vascular diseases (34–36) was investigated. In contrast to the known pro-inflammatory functions of IL-33, IL-33/ST2 signaling protected animals from experimentally induced cardiac failure by antagonizing angiotensin II-induced cardiomyocyte hypertrophy (110). Furthermore, IL-33 also dictates healing processes indirectly *via* ILC2s.

Under physiological conditions, ILCs reside in heart and display a progenitor-like phenotype (111). These heart resident ILCs are evident in biopsy samples from animals and humans with ischemic cardiomyopathy and myocarditis and are fated to differentiate to ILC2s in response to cardiac failure (111). ILC2s in pericardial adipose tissue (PcAT) proliferate in an IL-33 dependent manner in response to MI, and animals deficient in ILC2 exhibited incomplete recovery from heart dysfunction and a worsened mortality rate post-MI (112). Although the precise mechanism of ILC2s is unknown, the recruitment of eosinophils by IL-5 is considered in the recovery of cardiac function. This is supported by the observation that animals deficient in eosinophils failed to ameliorate cardiac functions after MI and that IL-4 from eosinophils was essential for recovery (113). However, the infiltration of eosinophils into heart needs to be regulated in order to avoid eosinophilia which induces inflammatory dilated cardiomyopathy (114).

Interestingly, low-dose IL-2 (aldesleukin) administration in patients with acute coronary syndrome exhibited transient activation of blood ILC2s, with a concomitant increase in serum IL-5 and eosinophil counts, demonstrating recovery of cardiac function (112). Further research on ILC2s in cardiac diseases will provide beneficial insights into developing unprecedented therapies.

### Protective Roles of ILC2s in Atherosclerosis

Atherosclerosis is an arterial disease characterized by the deposition of plaques on inner walls; and lipid modifications in plaques result in the generation of non-self-antigens, causing chronic inflammation. Atherosclerosis is the primary cause of most cardiovascular diseases. Administration of cytokines related to ILC2 activation were effective in reducing atherosclerosis in animals (115). TSLP (116), IL-25 (117), and IL-33 showed protective effects, and the effectiveness of IL-33 was largely dependent on IL-5 (118). ILC2s that were experimentally expanded with IL-2/IL-2R complexes protected from the development of atherosclerosis, although, the contribution of IL-5/eosinophils was limited (119). In contrast, ILC1 and NK cells were shown to play etiologic roles in disease development (120). This correlated well with a significantly high ILC1/ILC2 ratio in patients with acute cerebral infarction, commonly caused by rupture of atherosclerotic plaques (121). By selectively depleting ILC2s in an animal model of atherosclerosis, regional ILC2s that were in proximity to atherosclerotic lesions, sufficiently reduced atherosclerosis, possibly through phenotypic alteration of macrophages to anti-inflammatory M2 macrophages (122). Furthermore, transfer of ILC2s into mice that developed atherosclerosis led to an increase in B1 cell-derived atheroprotective IgM antibodies with reduction in plaque deposition (123). Collectively, ILC2s appear to be protective against atherosclerosis.

### Etiologic Roles of ILC2s in Pathogenesis of Pulmonary Arteries

In contrast to the protective roles of ILC2s in cardiac failure and atherosclerosis, chronic inflammation in lungs possibly drives ILC2s to act in mediating disorders of blood vessels, including pulmonary arterial hypertension (PAH). PAH is a progressive vascular disease characterized by a severe obstruction such as hypertrophy of small pulmonary arteries with high pulmonary arterial pressure, thereby resulting in right ventricular failure. It is categorized as one of the five groups of clinical classification for pulmonary hypertension (PH) (124). PAH is an intractable rare disease and its development is multifactorial. Although the investigation of causative factors of PAH is ongoing, chronic inflammation may have a plausible role in the underlying mechanism (125). Evidence suggests chronic allergic conditions in mice, concomitant with eosinophilia, lead to the induction of vessel remodeling with remarkable collagen deposition and enhanced proliferation of α-smooth muscle cells (126, 127). Subsequently, Th2 cytokines (128) or IL-5 and eosinophils (129) were reported to be necessary for the initiation of arterial remodeling. In humans, parasitic infection, in which Th2 cytokines such as IL-4 and IL-13 are predominant, is believed to be the most common cause of PAH (130, 131), with Th2 cytokines inducing arterial hypertrophy and other arterial modifications (131, 132).

Pulmonary arterial hypertrophy can also be experimentally induced by prolonged administration of IL-33 in mice (133). Histological examination revealed that perivascular ILC2s and eosinophils were evident around hypertrophied arteries, and this hypertrophy was ameliorated with anti-IL-5Rα mAb that depleted eosinophils (134). The proximity of ILC2s to blood vessels in lungs, as visualized in collagen-rich (135) adventitial niches (136), may facilitate their vascular regulation through eosinophil recruitment. In this region, ILC2s are maintained by IL-33-expressing stromal cells (136) which possibly regulate ILC2s in case of arterial hypertrophy. Thus, elucidation of the precise regulatory mechanism will help to understand the initial phase of disease development.

Because of the lack of histological evidence in humans on initial phase of arterial hypertrophy, animal models of PAH are essential to reveal causative factors. Despite reports of advanced arterial hypertrophy in animal studies, severe PH or right ventricular hypertrophy is not evident (101, 102, 105). The establishment of animal models that are more relevant to human PAH will not only help us to understand the underlying mechanism but is also imperative in developing a therapeutic strategy.

## Discussion/Conclusion

**Figure 1:**
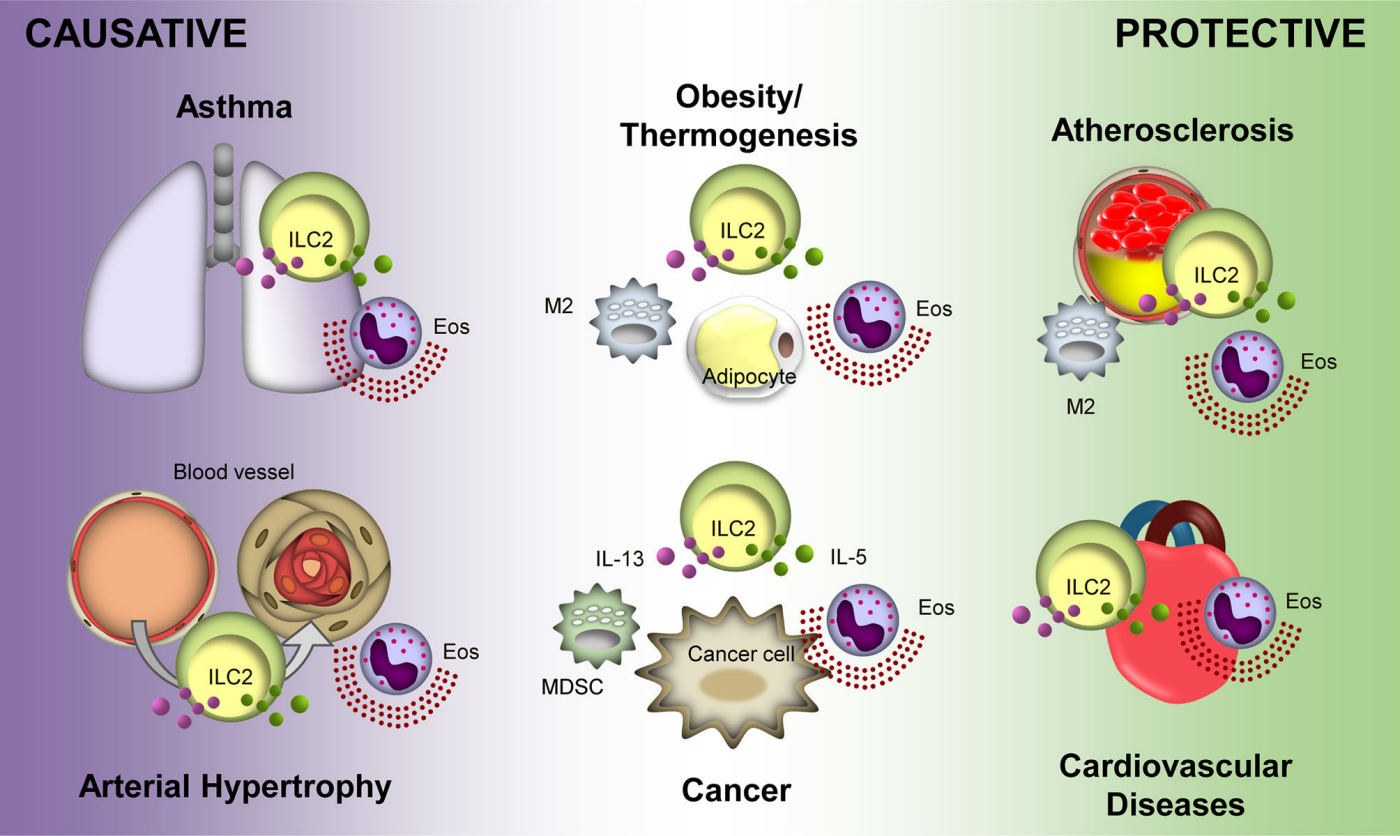
Protective and causative roles of Group 2 Innate Lymphoid Cells (ILC2s) in different diseases. ILC2s display a protective role in atherosclerosis and cardiovascular diseases. Overall, ILC2s is protective in obesity and thermogenesis; however, distal ILC2s may cause obesity. Conversely, ILC2s potentiate asthma and arterial hypertrophy. Their roles in cancer are varied depending on the tumor microenvironment and type of cancer. Purple and green particles depicted in the figure are interluekin-13 (IL-13) and IL-5, respectively. Eos, eosinophil; M2, M2 macrophage; MDSC, myeloid-derived supressor cell.

Recent advances in ILC2 research have revealed their pleiotropic roles in various diseases (**Figure 1**). Due to heterogeneity in the function of ILC2s in various disease conditions, their clinical application faces many obstacles. A treatment that targets ILC2s in one disease may be detrimental to another. For example, therapy for obesity by activating ILC2s with low doses of IL-2 may result in excess amounts of IL-5 from the ILC2s and facilitate tumor metastasis (89). These may present a similar effect in related diseases such as atherosclerosis (119) and MI (112). Thus, understanding the precise action of ILC2s in a particular disease and the extent of its effect on other diseases is indispensable. Delicate procedures for regulating ILC2s are required in addition to careful analyses of experimental and clinical observations, which will ultimately lead to efficient therapeutic regimes.

## Author Contributions

MI designed and wrote the manuscript. SN reviewed and revised the manuscript prior to submission. All authors have read and approved the final version of the manuscript.

## Funding

This work was supported by a Grant-in-Aid for Scientific Research (C) (Grant Number 19K07632 to MI) and (B) (21H02963 to SN) from the Japan Society for the Promotion of Science, and Precursory Research for Embryonic Science and Technology, Japan Science and Technology Agency (JPMJPR18H6 to SN).

## Conflict of Interest

The authors declare that the research was conducted in the absence of any commercial or financial relationships that could be construed as a potential conflict of interest.

## Publisher’s Note

All claims expressed in this article are solely those of the authors and do not necessarily represent those of their affiliated organizations, or those of the publisher, the editors and the reviewers. Any product that may be evaluated in this article, or claim that may be made by its manufacturer, is not guaranteed or endorsed by the publisher.
